# Supplementary figures and images for: Uncovering hidden genetic risk factors for breast and ovarian cancers in BRCA-negative women: a machine learning approach in the Saudi population
Source: PeerJ Comput Sci. 2024 Apr 19;10:e1942. doi: 10.7717/peerj-cs.1942 (PMC11042021; doi:10.7717/peerj-cs.1942)

Figure 1 : GO cellular component analysis of gene associated with BRCA-related cancer.


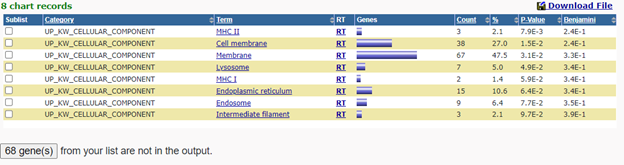

Supplement: Supplemental Information 1 [file peerj-cs-10-1942-s001.docx]

Figure 2 : GO biological processes analysis of genes associated with BRCA-related cancer.


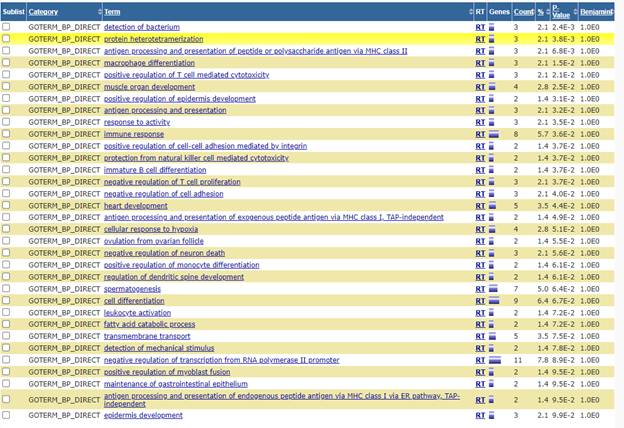

Supplement: Supplemental Information 2 [file peerj-cs-10-1942-s002.docx]

Figure 3 : GO molecular function analysis of genes associated with BRCA-related cancer.


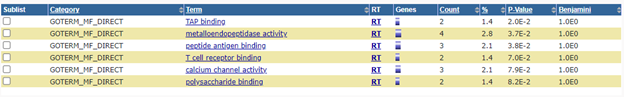

Supplement: Supplemental Information 3 [file peerj-cs-10-1942-s003.docx]
